# Engineering 3D bicontinuous hierarchically macro-mesoporous LiFePO_4_/C nanocomposite for lithium storage with high rate capability and long cycle stability

**DOI:** 10.1038/srep25942

**Published:** 2016-05-16

**Authors:** Qian Zhang, Shao-Zhuan Huang, Jun Jin, Jing Liu, Yu Li, Hong-En Wang, Li-Hua Chen, Bin-Jie Wang, Bao-Lian Su

**Affiliations:** 1 Laboratory of Living Materials at the State Key Laboratory of Advanced Technology for Materials Synthesis and Processing, Wuhan University of Technology, 122 Luoshi Road, 430070, Wuhan, Hubei, China; 2 FEI company, Shanghai Nanoport, 399 Shenxia Road, 201210 Shanghai, China; 3 Laboratory of Inorganic Materials Chemistry (CMI), University of Namur, 61 rue de Bruxelles, B-5000 Namur, Belgium; 4 Department of Chemistry and Clare Hall, University of Cambridge, Cambridge, CB2 1EW, United Kingdom

## Abstract

A highly crystalline three dimensional (3D) bicontinuous hierarchically macro-mesoporous LiFePO_4_/C nanocomposite constructed by nanoparticles in the range of 50~100 nm via a rapid microwave assisted solvothermal process followed by carbon coating have been synthesized as cathode material for high performance lithium-ion batteries. The abundant 3D macropores allow better penetration of electrolyte to promote Li^+^ diffusion, the mesopores provide more electrochemical reaction sites and the carbon layers outside LiFePO_4_ nanoparticles increase the electrical conductivity, thus ultimately facilitating reverse reaction of Fe^3+^ to Fe^2+^ and alleviating electrode polarization. In addition, the particle size in nanoscale can provide short diffusion lengths for the Li^+^ intercalation-deintercalation. As a result, the 3D macro-mesoporous nanosized LiFePO_4_/C electrode exhibits excellent rate capability (129.1 mA h/g at 2 C; 110.9 mA h/g at 10 C) and cycling stability (87.2% capacity retention at 2 C after 1000 cycles, 76.3% at 5 C after 500 cycles and 87.8% at 10 C after 500 cycles, respectively), which are much better than many reported LiFePO_4_/C structures. Our demonstration here offers the opportunity to develop nanoscaled hierarchically porous LiFePO_4_/C structures for high performance lithium-ion batteries through microwave assisted solvothermal method.

Lithium-ion batteries (LIBs) have been rapidly developed for applications in plug-in hybrid electric vehicles (HEVs), electric vehicles (EVs) and large-scale energy storage due to their high energy density and durable cycle life^1^,^2^,^3^,^4^,^5^,^6^. In general, the performance of LIBs is determined by the electrode materials. From the viewpoint of electrode materials, the olivine-type LiFePO_4_ is considered as one of the most promising cathode materials owing to its high operating voltage (~3.4 V vs Li/Li^+^), high theoretical capacity (~170 mA h/g), low cost and environmentally benign^7^,^8^. In fact, LiFePO_4_ has been successfully used for HEVs and EVs. The low intrinsic electronic^9^ and ionic conductivity^10^ of LiFePO_4_, however, limit its widespread applications. Such poor electronic conductivity is caused by the lack of mixed valence due to the low solubility of LiFePO_4_ and FePO_4_ and the highly localized Fe^2+^ or Fe^3+^ ions, while the poor Li^+^ conductivity is caused by one-dimensional diffusion of Li^+^ to form edge-shared LiO_6_ octahedron along b-axis^11^. Thus, seeking approaches to improve its electrochemical performance is still highly pursued by materials scientists.

At present, huge efforts have been made to address the above problems, including reducing the particle size^12^,^13^,^14^ to shorten the ionic and electrical path length, and coating carbon or other conducting layers^15^,^16^,^17^ to enhance the electrical conductivity. Combination of nanostructure with carbon coating is a widely adopted route to effectively resolve the aforementioned low intrinsic electronic and ionic conductivity problem^18^,^19^,^20^. According to the characteristic time constant t for diffusion being proportional to the square of diffusion length L (t ≈ L^2^/D)^21^, one can see that reducing the characteristic dimensions of electrolytically active materials is more effective to improve battery cycling rates than increasing the ion diffusivity D. However, due to the inadequate contact between the electrodes and electrolytes, single structured nanomaterials such as nanoparticles^22^, nanorods^23^, nanowires^24^ are difficult, to some extent to obtain the highly efficient ion and electron pathways. Recently, our group found that hierarchically nanostructured porous materials, benefiting from large surface areas for reaction, interfacial transport, or dispersion of active sites at different length scales of pores and shortened diffusion paths or reduced diffusion effect, can largely improve their electrochemical properties^25^,^26^,^27^,^28^,^29^,^30^. Preparation of hierarchically porous LiFePO_4_ nanostructures is thus a promising route to improve their electrochemical properties, such as capacities, rate performances and cycle life.

Among the various synthesis methods^18^,^31^,^32^,^33^,^34^, hydrothermal methods have been particularly successful in offering high performance LiFePO_4_ nanostructures^35^,^36^,^37^. But long reaction time is often required. In our previous work, we found that the microwave assisted hydrothermal method can quickly enhance the crystallization of nanoparticles constructed mesoporous TiO_2_ microspheres for high performance LIBs^38^. This means microwave assisted hydrothermal synthesis may not only improve the crystallization of the target materials but also short the reaction time. In fact, microwave assisted hydrothermal synthesis has been adopted to rapidly synthesize products with highly controllable particle size and morphology^39^,^40^. However, to the best of our knowledge, synthesis of hierarchically porous LiFePO_4_ nanostructures via microwave assisted solvothermal method has never been reported.

Herein, we report the synthesis of highly crystalline 3D bicontinuous hierarchically macro-mesoporous LiFePO_4_/C (LFP/C) nanocomposite as cathode material for LIBs. We first synthesize the LiFePO_4_ precursor (LFP-P) via a rapid microwave assisted solvothermal process in 1 h. To increase the conductivity of the as-prepared LFP-P, sucrose is then used as carbon source to coat carbon layer on the surface. After calcination at 700 °C, the highly crystalline 3D hierarchically macro-mesoporous LFP/C nanocomposite is obtained. The as-synthesized LiFePO_4_/C cathode exhibits low polarization, enhanced electrical conductivity, excellent rate capability and long cycling life for LIBs.

## Results

**Figure 1:**
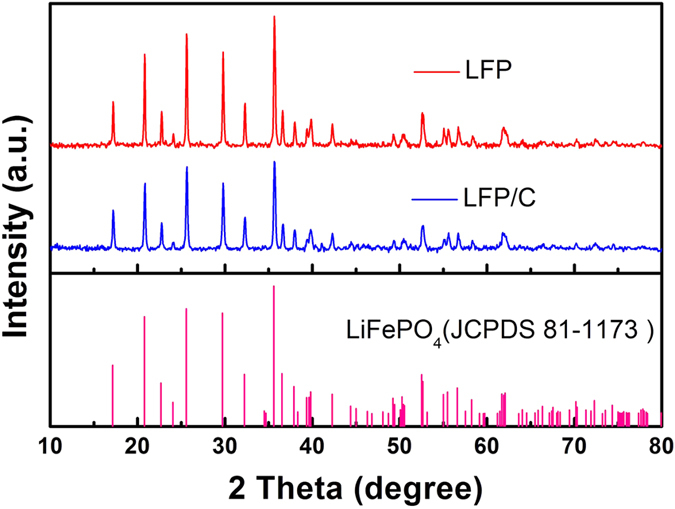
XRD patterns of the as-synthesized LFP and LFP/C.

X-ray diffraction (XRD) patterns of the as-synthesized LFP and LFP/C materials are shown in Fig. 1. The entire diffractions of the two samples match well with the standard orthorhombic LiFePO_4_ (JCPDS: 81–1173). The strong and sharp peaks suggest good crystallinity for two samples, which is quite different from the XRD patterns of LFP-P (Figure S1). It is noticeable that the peaks of LFP/C are wider than that of LFP, indicating smaller nanoparticles in LFP/C compared with LFP. No impurities such as Li_3_PO_4_ can be observed, suggesting that our method is favorable for pure LiFePO_4_ preparation. No typical diffraction peaks of carbon are found in LFP/C sample, indicating the carbon in amorphous form.

**Figure 2:**
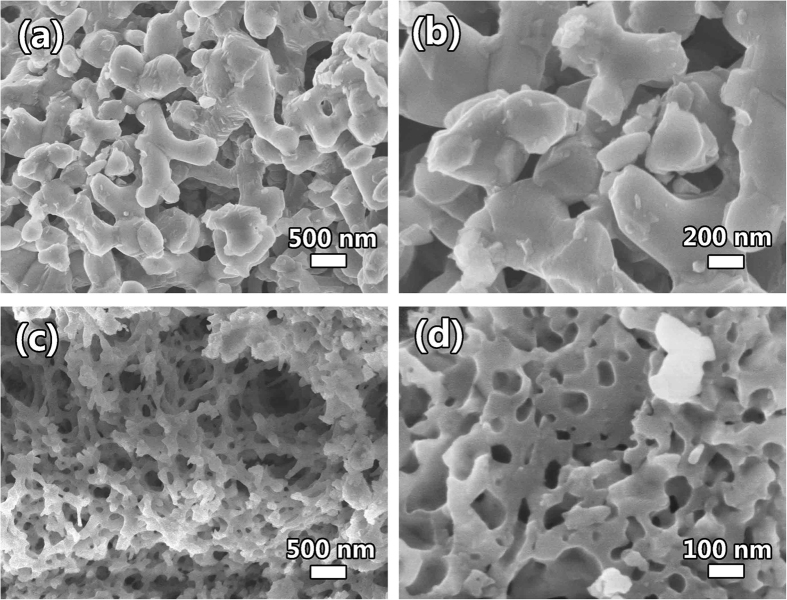
SEM images of the as-synthesized (**a,b**) LFP and (**c,d**) LFP/C.

The morphologies of the as-synthesized LFP-P, LFP and LFP/C samples are observed by scanning electron microscopy (SEM). Figure S2 illustrates the morphology of LFP-P, which gives a 3D bicontinuous macroporous structure constructed by nanoparticles of ~30 nm. Noticeably, after calcination, the macroporous structure is well retained as shown in Fig. 2a. However, the nanoparticles of LFP grow to ~300 nm (Fig. 2b). After carbon coating, the 3D hierarchically macroporous structure is still maintained (Fig. 2c). Figures S3–S5 present more SEM images to show the hierarchically porous structure among the whole structure. Figure 2d also shows that the nanoparticles are jointed each other to maintain 3D bicontinuous hierarchically macro-mesoporous structure instead of aggregation together. It is noted that after carbon coating, the nanoparticles of LFP/C can keep their small size at 50~100 nm. This means that the carbon coating can effectively prevent the quick growth of LiFePO_4_ nanoparticles, which is quite important to shorten the ionic and electrical path lengths for LIBs.

**Figure 3:**
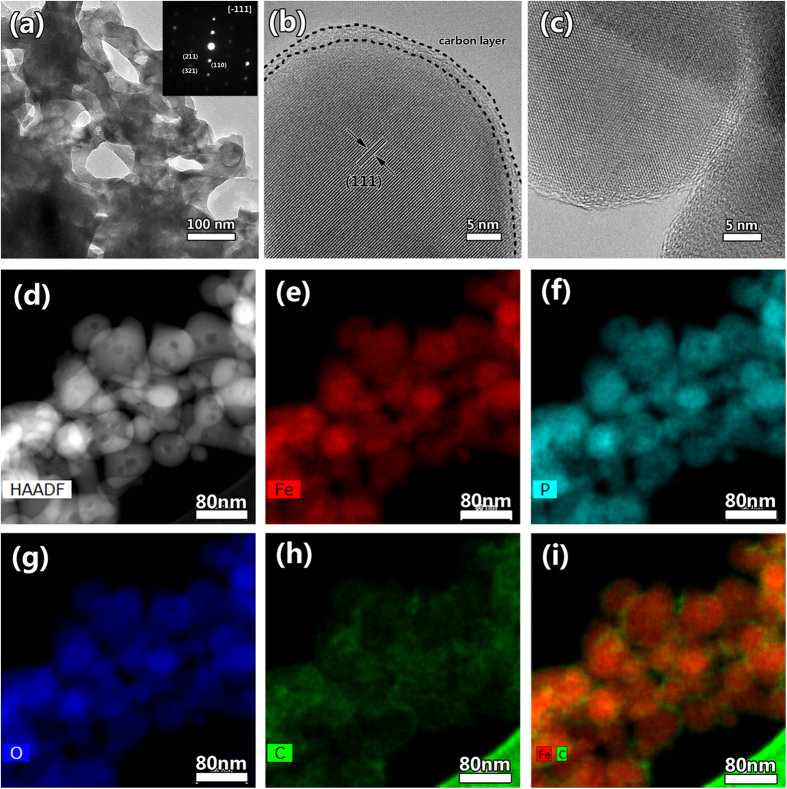
(**a**) TEM image, (**b,c**) HRTEM images and (**d–i**) STEM-EDS elemental mapping images of LFP/C. The insert in (**a**) is the SAED pattern from one nanoparticle.

The TEM characterizations are employed to reveal more detail structure information of LFP and LFP/C. Figure S6 presents the TEM and HRTEM images of LFP. It clearly shows the bicontinuous macroporous structure constructed by nanoparticles and no amorphous layer can be found at the surface of the particle. Figure 3a displays the typical TEM image of LFP/C, indicating the macro-mesoporous structure. This is consistent with SEM observation. It is noted that there are also many pores inside the nanoparticles (Fig. 3a and Figure S7). This is also clearly displayed in HAADF image (Fig. 3d). Such 3D bicontinuous macro-mesoporous structure constructed by highly crystalline LFP nanoparticles is very helpful for LIBs, which can facilitate the electrolyte permeation and the smaller nanoparticles can provide short diffusion lengths for Li^+^ in the intercalation-deintercalation process. The inserted SAED pattern in Fig. 3a is recorded from one nanoparticle. The sharp diffraction spots indicate the nanoparticles are single crystals. This is also useful for electrons and ions diffusion in the crystal structure. The HRTEM in Fig. 3b demonstrates that the lattice fringes correspond to the (111) crystal plane of olivine-type LiFePO_4_. A uniform carbon layer at ~3 nm on the LiFePO_4_ crystal surface can be clearly observed. This thin and uniform coated carbon layer can smooth electron migration for the reverse reaction of Fe^3+^ to Fe^2+^. Figure 3c displays the interface of two nanoparticles. It clearly shows that there is no obvious interface between two nanoparticles, verifying that the nanoparticles are interconnected each other. This verifies that the nanoparticles are jointed each other to form 3D bicontinuous hierarchically macro-mesoporous structure. To clearly reveal the element distribution in LFP/C, STEM-EDS technique is employed as shown in Fig. 3d–i. The element mapping images from Fig. 3e–g show the homogeneous distribution of Fe, P and O elements among the whole product. In particular, Fig. 3h displays the carbon layer in LFP/C, demonstrating the hollow structure of carbon. After the super imposition of images about Fe and C together, Fig. 3i obviously shows that the carbon is at the shell of LiFePO_4_ nanoparticles, verifying C uniformly coated on the surface of LiFePO_4_ nanoparticles.

**Figure 4:**
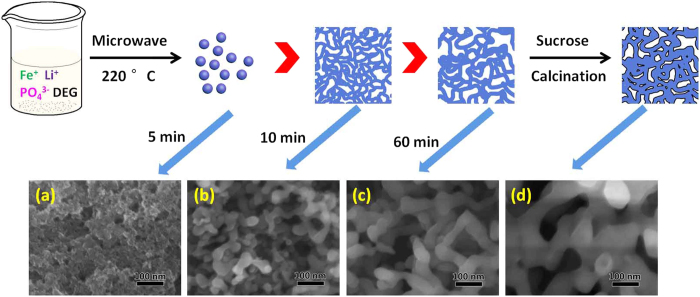
Schematic illustration for the preparation of LFP/C. The embedded SEM images are the products at (**a**) 5 min, (**b**) 10 min, (**c**) 60 min and (**d**) LFP/C.

The growth process of such 3D bicontinuous macro-mesoporous structure is monitored and schemed in Fig. 4. We can see that when the reaction time is 5 minutes, the products are small nanoparticles with size of ~10 nm. After 10 minutes, the nanoparticles further grow and begin to assemble to form a 3D bicontinuous macro-mesoporous structure. And the following 50 minutes is just the crystallites further growth and crystallization, ultimately to form LFP-P. After sucrose is added accompanying with the subsequent calcination, the nanoparticles are coated with a carbon layer and the 3D bicontinuous hierarchically macro-mesoporous LiFePO_4_/C nanocomposite is formed.

**Figure 5:**
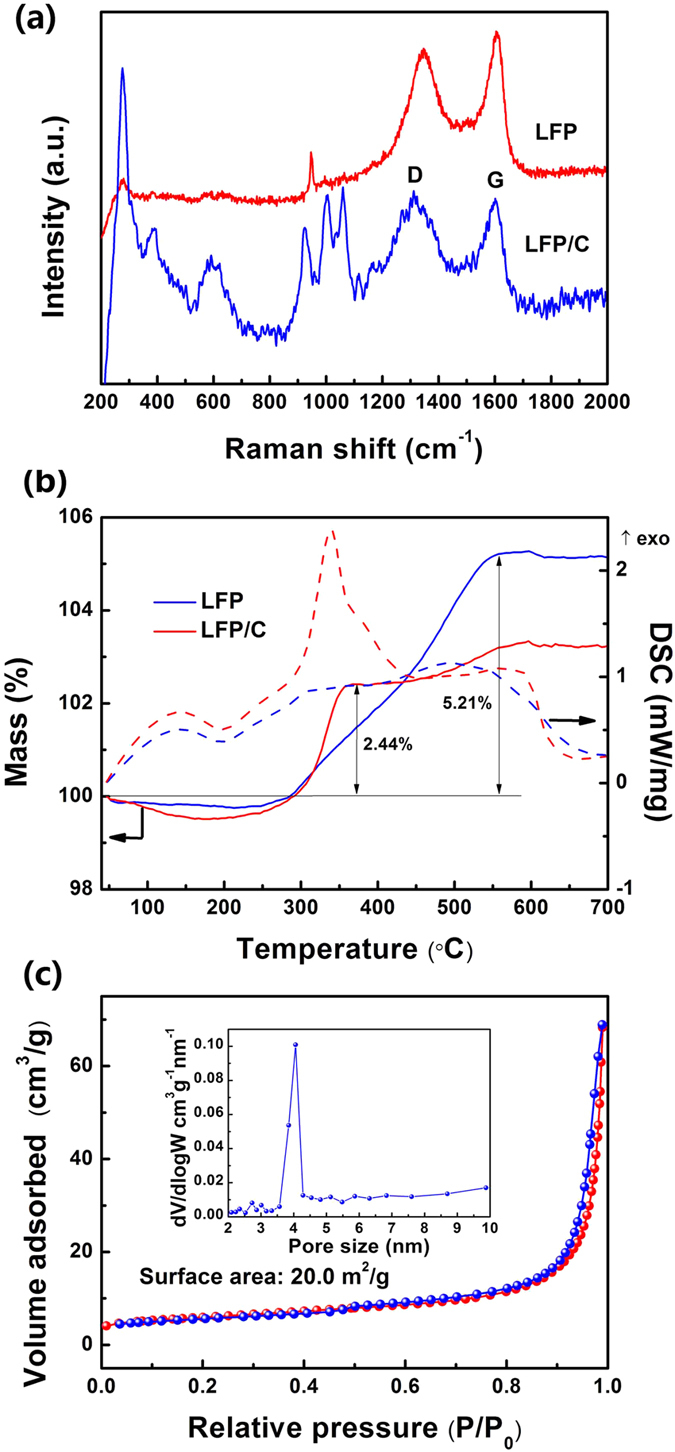
(**a**) Raman spectra of LFP and LFP/C, (**b**) TG-DSC curves of the LFP and LFP/C recorded from the room temperature to 900 °C at a heating rate of 10 °C min^−1^ in air, (**c**) Nitrogen adsorption-desorption isotherm and the corresponding pore size distribution (inset) of the LFP/C composite.

Raman characterization is performed to probe the vibrational modes of both crystalline and amorphous materials in LFP and LFP/C (Fig. 5a). The Raman spectrum of LFP/C displays two broad peaks at 1320 and 1609 cm^−1^, corresponding to the D band (disordered carbon, sp^3^) and G band (graphite, sp^2^) of Raman vibration modes for amorphous carbon, respectively^41^,^42^,^43^,^44^,^45^. The D band and G band are also been observed for LFP, indicating carbon existence although it is not observed by TEM. This should be originated from the carbonization of the absorbed DEG molecules on LFP-P, resulting in very thin carbon layer or randomly distributed carbon in LFP. It is interesting to see that the I_D_/I_G_ ratio (0.92) of LFP is a little lower than that of LFP/C (1.02), indicating less carbon source is more suitable for organic molecules carbonization. However, too low carbon content is not helpful for particle size control according to our SEM and TEM observations. Other peaks should be attributed to the orthorhombic LiFePO_4_. Particularly, two peaks at 947 cm^−1^ and 586 cm^−1^ for both samples are assigned to the intra molecular stretching modes (ν1, ν4) of the PO_4_^3−^ anion^43^. The Raman behaviors are very well consistent with those already reported LiFePO_4_/C composites^18^,^46^,^47^. It is noted that the signals of LFP/C are higher than those of LFP. This should be close related to the better crystal structure features of LFP/C comparing to LFP, which is very important for electrons and ions diffusion in the structure leading to better electrochemical performance of LFP/C.

TG/DSC measurement of LFP and LFP/C is conducted to estimate the carbon content (Fig. 5b). For LFP, a weight gain around 5.21 wt% at 250–500 °C corresponds to the oxidation of LiFePO_4_ to Li_3_Fe_2_(PO_4_)_3_ and Fe_2_O_3_^31^. For LFP/C, the weight gain is only 2.44 wt%, due to the oxidation of carbon in the composite (an apparent exothermic peak around 325 °C), leading to a slight decrease of weight gain. Then the carbon content is 2.77 wt% for LFP/C. Above the temperature of 600 °C, a total oxidization of both LiFePO_4_ and carbon is completed.

The nitrogen adsorption-desorption isotherm of LFP/C exhibits a type-II shape (Fig. 5c), indicating the presence of macropores^26^. LFP/C exhibits a BET specific surface area of 20.0 m^2^/g, much higher than that of LFP (0.1 m^2^/g). This high BET surface area is favorable for enhanced contacting with the liquid electrolyte. Moreover, the pore size distribution (Fig. 5c inset) gives pore size centered at ~4 nm. The presence of 3D hierarchical macro-mesoporosity is beneficial for electrolyte ions diffusion and transport through the LFP/C composite.

**Figure 6:**
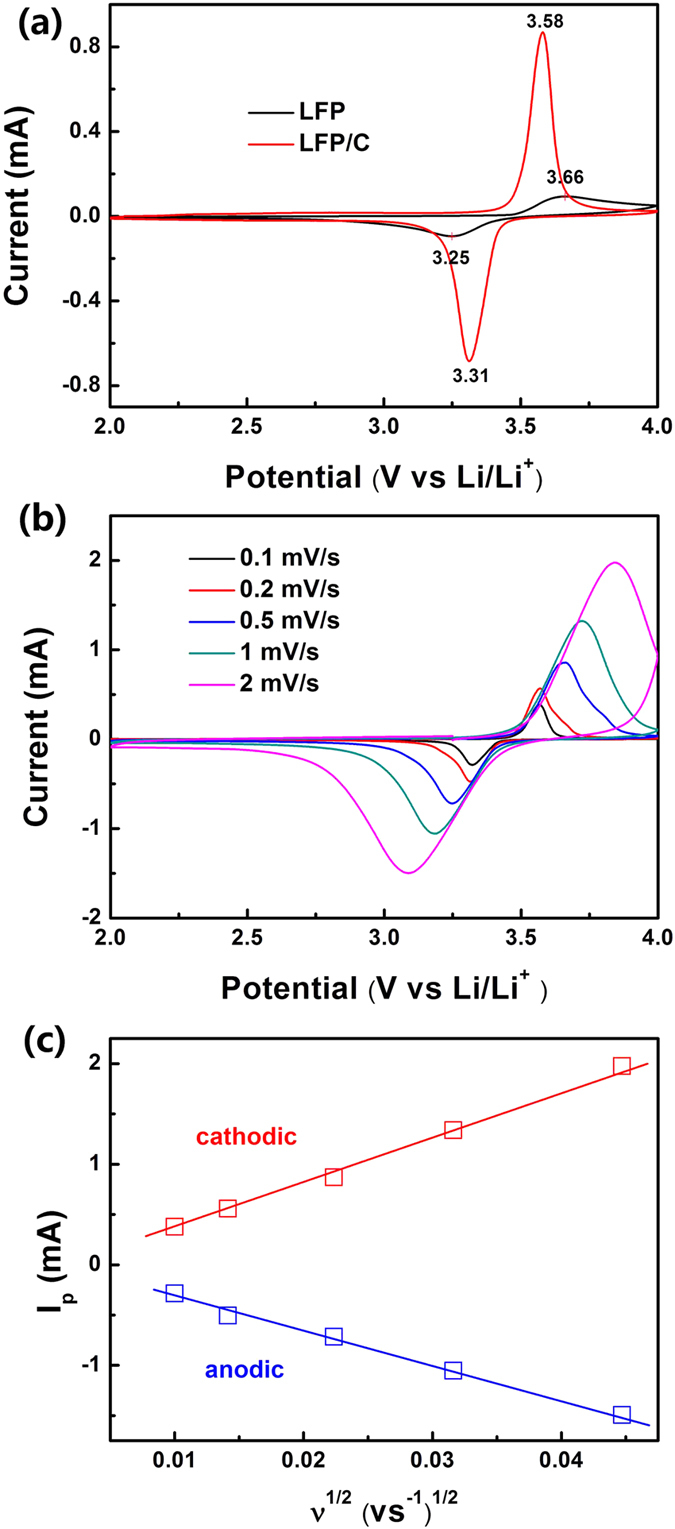
(**a**) CV curves of LFP and LFP/C electrodes at a scan rate of 0.2 mV/s, (**b**) CV curves of the LFP/C electrode at scan rates of 0.1~2 mV/s, (**c**) linear response of the peak current (I_p_) as a function of the square root of scanning rate (ν).

Figure 6a presents the cyclic voltammograms (CV) curves of the LFP/C and LFP electrodes at a scan rate of 0.2 mV/s within the potential window of 2–4 V (vs. Li/Li^+^). For the LFP/C electrode, the anodic peak at 3.58 V corresponds to the oxidation of Fe^2+^ to Fe^3+^, while the cathodic one appearing at 3.31 V is associated with the reduction of Fe^3+^ to Fe^2+ ^48. The potential interval between the two redox peaks is 0.27 V. The narrow peak separation means a low polarization of the electrodes, indicating the easily electrochemical reverse reaction of Fe^3+^ to Fe^2+^ during the Li^+^ insertion-desertion process. As for the LFP electrode, the potential interval between the two redox peaks is 0.41 V, much larger than that of LFP/C. This can lead to a restricted electrochemical reverse reaction from Fe^3+^ to Fe^2+^ during the Li^+^ insertion-desertion process. This phenomenon means that the carbon layer can improve the electronical conductivity of the LFP/C electrode, resulting in a decreased polarization of the electrode. In addition, the peak profiles of LFP/C are narrower with a high peak current, indicating a lower conductivity restriction and diffusion limitation comparing to LFP. These results highlight the improvements of LFP/C for high performance of LIBs that come from the 3D bicontinuous macro-mesoporous structure, the small particle size and uniformly coated carbon layer.

Figure 6b shows the CV results of LFP/C electrode at various scanning rates from 0.1–2.0 mV/s after a scan rate of 0.2 mV/s for three cycles. The symmetry of the sharp oxidation and reduction peaks confirms the good reversibility of lithium extraction-insertion reactions in LFP/C. Two redox peaks are observed between 3.3 V and 3.6 V (vs. Li/Li^+^) at a scan rate of 0.1 mV/s. These well-defined peaks correspond to the insertion and de-insertion of Li^+^ in the LiFePO_4_ nanoparticles and are still clearly visible at higher scanning rates with wider separation of peak positions. The peak currents I_p_ (Amperes) at different sweep rates can be used to evaluate Li^+^ diffusion coefficient D (cm^2^/s), according to the Randles Sevcik equation^49^: I_p_ = 2.69 × 10^5^ A C D^1/2^ n^3/2^ v^1/2^, where A is the electrode area (cm^2^), C is the concentration of the species being oxidized or reduced (mol/cm^3^), n is the number of electrons transferred (n = 1 for Fe^2+^/Fe^3+^ redox pair), and v is the potential scan rate (V/s). The peak current is in linear response to the square root of scanning rate (ν) as shown in Fig. 6c. The average Li^+^ diffusion coefficients of the LFP/C composite are estimated to be ~7.0 × 10^−14^ cm^2^ s^−1^ and ~4.0 × 10^−14^ cm^2^ s^−1^ for the charge and discharge processes respectively which are as good as the nano-sized LiFePO_4_ material reported before^50^.

**Figure 7:**
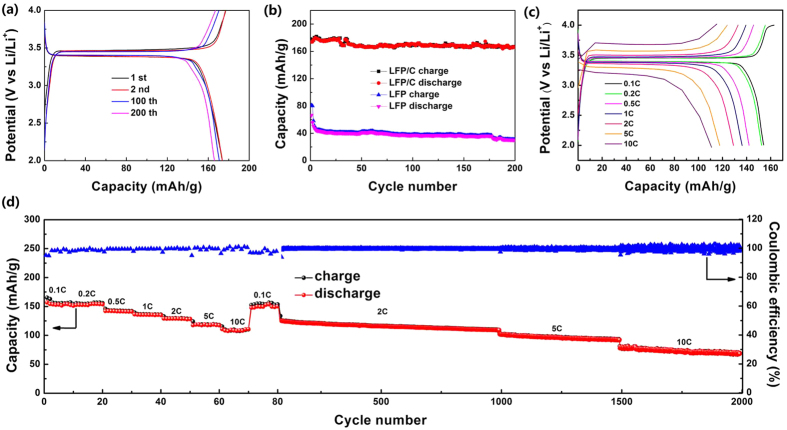
(**a**) Charge and discharge profiles of LFP/C between 2.0 and 4.0 V at 0.2 C for the first, second, 100th and 200th cycles. (**b**) Cycle performance of LFP and LFP/C at 0.2 C. (**c**) Charge and discharge profiles of LFP/C in the potential region from 2.0 to 4.0 V at various rates. (**d**) The long-cycle rate performances of LFP/C.

The typical charge and discharge profiles of the LFP/C electrode at a current rate of 0.2 C are shown in Fig. 7a. The LFP/C electrode exhibits the high first charge and discharge capacities of 177.1 and 173.8 mA h/g respectively, with an initial coulombic efficiency of 98%, and the second cycle curves match well with the initial ones, and after 100 and 200 cycles, the capacity retention is 98.3% and 95.6% respectively, indicating the excellent cyclic stability. The coated carbon layer and the small particle size increase the electrical and ionic conductivity and further enhance the kinetic reaction, facilitating more Fe^2+^ oxidation to Fe^3+^ as suggested by CV results, leading to higher capacity than LFP. It is noticeable that the profiles of LFP/C are long and flat, and the gap between the charge and discharge curves of LFP/C is small, indicating the fast redox reaction and phase transition in LFP/C. Such excellent performance of LFP/C is attributed to the 3D bicontinuous macro-mesoporous structure, small particle size and coated carbon layer, which ensure the short transport of Li^+^ without excessive polarization and improved electrical and ionic conductivity.

Figure 7b presents the cycle performance of the LFP/C and LFP electrodes at 0.2 C. The initial discharge capacity of LFP/C and LFP is 173.8 and 66.6 mA h/g, and after 200 cycles, the capacity retention is 95.6% and 45.6%, respectively. The results demonstrate the excellent cycling stability of the 3D macro-mesoporous LFP/C nanocomposite.

Figure 7c shows the charge-discharge curves at various discharge rates from 0.1 C to 10 C of LFP/C. The flat voltage plateaus around 3.4 V imply the two-phase LiFePO_4_ ↔ FePO_4_ + Li^+^ + e^−^ reaction^7^. The voltage differences between the charge and discharge curves at 0.1, 1, 5 and 10 C (measured at the half capacities of these curves) are ~63, 104, 294.5 and 518 mV, respectively. The slight voltage differences indicate the good electronic conductivity of the LFP/C nanocomposite, resulting in the better redox reactions in LFP/C during the Li^+^ insertion-desertion process. This is in agreement with the CV results.

Figure 7d gives the rate performance of the LFP/C electrode. As can be seen, the capacity values drop with the increase of charge-discharge rates, being associated with the sluggish Li^+^ diffusion kinetics at very high rates. As for LFP/C, a high discharge capacity of 156.9 mA h/g is achieved at a low rate of 0.1 C. A discharge capacity of 129.1 mA h/g can be obtained at 2 C. The capacity can still be maintained at 110.9 mA h/g after the rate increases to 10 C. When the current density returns back to 0.1 C, the discharge capacity recovers to 152.9 mA h/g, demonstrating the high rate performance and stability of LFP/C. The long-cycle rate performance of the LFP/C electrode is further employed at the same unit cell. It presents that even at the high rate of 5 C and 10 C, the LFP/C can still keep high and steady capacity. The capacity retention is 87.2% at 2 C (1000 cycles), 76.3% at 5 C (500 cycles) and 87.8% at 10 C (500 cycles). After 2000 cycles at different rates, the discharge capacity can still maintain at 80 mA h/g. The Coulombic efficiency is always ~100% during the whole process.

The rate performance of our LFP/C composite is much better than nanostructured LiFePO_4_/C^20^, hierarchically dumbbell-like LiFePO_4_ microstructures^51^, LiFePO_4_ microstructures^52^ and rugby-like LFP/C/RGO^53^, almost the same with monodisperse hollow LiFePO_4_ microspheres^54^ and hierarchically structured LiFePO_4_^55^. This result indicates that our LFP/C composite exhibits a competitive rate performance than most of the previous results, owing to the 3D bicontinuous hierarchically macro-mesoporous structure, the small particle size and coated carbon layer. Therefore, it can be regarded that the 3D bicontinuous hierarchically macro-mesoporous LFP/C composite developed in this work provides a high rate capability.

**Figure 8:**
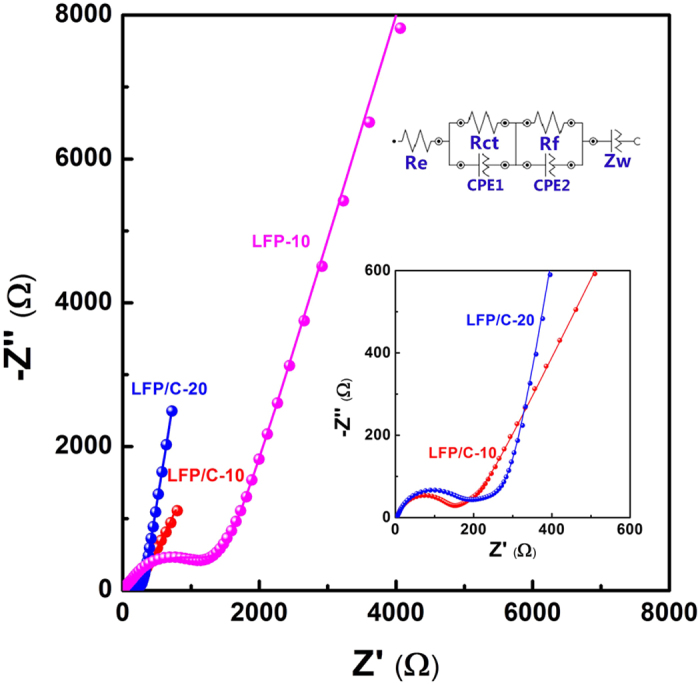
The equivalent circuit and Nyquist plots of the LFP and LFP/C electrodes. Frequency range: 10 mHz–100 kHz.

The electrochemical impedance spectroscopy (EIS) of the LFP and LFP/C electrodes is performed to further investigate their electrochemical kinetics (Fig. 8). The kinetic differences of LFP/C electrode after 10 cycles (LFP/C-10), after 20 cycles (LFP/C-20) and LFP electrode after 10 cycles (LFP-10) are investigated by modeling AC impedance spectra based on the modified equivalent circuit. In the equivalent circuit, R_e_ is the total resistance of electrolyte, electrode and separator. CPE_1_ and R_ct_ are the double layer capacitance and charge transfer resistance, respectively. CPE_2_ and R_f_ are the capacitance and resistance of the surface film formed on the electrode, respectively. Z_w_ is the Warburg impedance related to the diffusion of lithium ions into the bulk electrode. The fitting values from this equivalent circuit are presented in Table 1. It clearly shows that the diameter of semicircle for the LFP/C electrode in the high medium frequency region is much smaller than that of the LFP electrode, suggesting that the LFP/C electrode possesses a lower charge transfer resistance. The value of the diameter of the semicircle on the real axis is approximately equal to R_ct_. The R_ct_ value of the LFP/C-10 electrode (108.6 Ω) is slightly lower than that of the LFP/C-20 electrode (131.3 Ω), which is related to the slight polarization during cycling process, but much lower than that of the LFP-10 electrode (1476.2 Ω). These results confirm that the carbon coating endows the LiFePO_4_ electrode with a high conductivity and largely enhanced electron transport during the electrochemical lithium insertion/extraction reaction.

**Figure 9:**
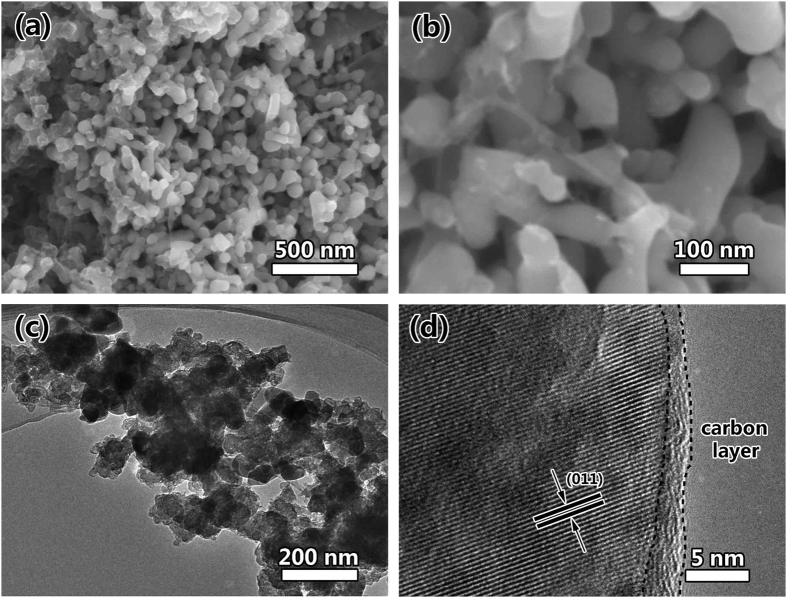
Post-mortem studies of the LFP/C cathode material after 2000 charge-discharge cycles at different rates. (**a,b**) SEM images, (**c**) TEM image and (**d**) HRTEM image.

To further understand the electrochemical performance and structural stability of the LFP/C cathode material, postmortem studies after 2000 charge-discharge cycles at different rates are carried out through SEM and TEM observations. For the post-mortem studies, the LFP/C cathode material after 2000 cycles is removed from the unit and immersed in acetone for one week to wash off the electrolyte. SEM (Fig. 9a,b) and TEM (Fig. 9c) images display that the 3D macroporous architecture is maintained after the electrochemical reaction, indicating the structural and electrochemical stability of LFP/C. This leads to the excellent capacity retention and superior rate performance. The HRTEM in Fig. 9d still shows the ~3 nm carbon layer on the LiFePO_4_ crystal surface. The lattice spacing of the particle is measured to be 3.64 Å, corresponding to the (011) crystal plane of LiFePO_4_. This result shows that the 3D bicontinuous hierarchically macro-mesoporous structure is retained well after the long electrochemical reaction at different high rates.

**Figure 10:**
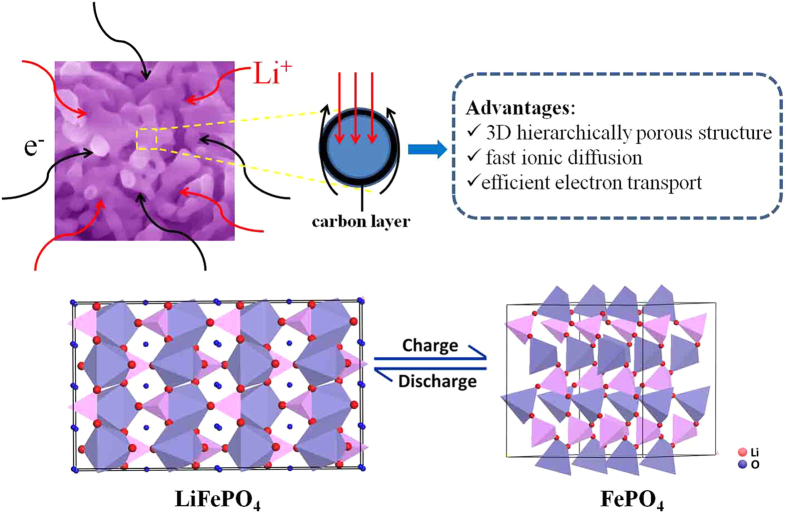
Mechanism diagram of LFP/C for Li^+^ intercalation-deintercalation.

According to the above results and discussion, the attractive high performance of LFP/C is achieved. This can be ascribed to a mixed conducting network with 3D bicontinuous hierarchical macro-mesopores serving as the ionic conducting network, the carbon coating on the surface of LiFePO_4_ crystals serving as the electronic conducting network, and the small nanoparticles providing short diffusion lengths for Li^+^ insertion-deinsertion as schemed in Fig. 10. The synergy of the above mentioned features leads to this 3D macro-mesoporous architecture of LFP/C being of the promising electrode material for high-power and high-energy lithium ion batteries.

## Discussion

Highly crystalline 3D bicontinuous macro-mesoporous LFP/C nanocomposite has been synthesized via a rapid microwave assisted solvothermal process and subsequent carbon coating. The as-synthesized LFP/C composite exhibits excellent electrochemical performance, especially for high rate performance. This can be ascribed to several factors, such as the 3D bincontinuous macro-mesoporous structure, the fast ionic diffusion in the crystalline nano-sized LiFePO_4_ as well as the efficient electron transport that benefits from the intimate contact between LiFePO_4_ nanoparticles and conductive carbon layer, all of which endow the high performance of LiFePO_4_. This work makes us believe that if we can ensure the *in-situ* carbonization to get the carbon layer with appropriate thickness (2~3 nm), this structure can bring even much better performance for LIBs.

## Methods

### Synthesis of LFP-P

In a typical synthesis, 0.01 mol Fe(NO_3_)_3_•9H_2_O and 0.01 mol CH_3_COOLi•2H_2_O are added into 70 mL diethylene glycol (DEG). After vigorously magnetic stirring for 0.5 h, a red wine-colored transparent solution is formed. Then 0.01 mol H_3_PO_4_ (85 wt% solution) is added into the above solution, and it changes to yellow instantly. After vigorously magnetic stirring for 0.5 h, the reaction solution is transferred into a 100 mL Teflon vessel, sealed, and heated at 220 °C for 1 h in a commercial microwave reaction apparatus (MDS-8G, Shanghai Sineo Microwave Chemistry Technology Co. Ltd.). After cooling down to room temperature, the obtained grey dark slurry is centrifuged, washed several times with absolute alcohol and distilled water, and finally dried at 60 °C for 12 h to get LiFePO_4_-precursor (LFP-P).

### Synthesis of LFP/C and LFP

The LFP/C composite is obtained by mixing LFP-P powder and sucrose (the weight ratio of LFP-P: Carbon = 1: 0.04) in 4 mL distilled water and 6 mL absolute alcohol. The mixture is stirred at 80 °C until the distilled water and absolute alcohol completely evaporated, and then dried at 60 °C for 12 h. The mixture is grinded and annealed at 700 °C under Ar/H_2_ (95:5) atmosphere for 10 h with a heating rate of 3 °C min^−1^. To obtain LiFePO_4_ (LFP), the LFP-P is directly annealed following the procedure described for LFP/C.

### Materials characterization

X-ray diffraction (XRD) patterns are obtained by a Bruker diffractometer at 40 kV, 40 mA, with Cu Kα radiation (λ = 1.54056 Å). The morphology of all the products is performed on scanning electron microscopy (SEM, Hitachi S-4800) equipped with a field-emission gun at an accelerated voltage at 5 kV. Transmission electron microscopy (TEM), high resolution transmission electron microscopy (HRTEM), scanning transmission electron microscopy (STEM) and energy-dispersive X-ray spectroscopy (EDS) are acquired on an FEI Talos F200X with an acceleration voltage of 200 kV. Thermogravimetric analysis (TGA) is performed using a Labsys Evo S60/58458 thermal analysis instrument at a temperature ramping rate of 5 °C min^−1^ in air atmosphere. Raman spectra are carried out at room temperature on an Invia Raman Microscope (Invia Microscope, Renishaw, UK) with 514.5 nm laser radiation at a laser power of 0.48 mW in the range of 200–2000 cm^−1^. Nitrogen adsorption–desorption isotherms were obtained using a Tri Star surface area & porosity analyzer (Tri Star II 3020) at 77 K. The specific surface area was calculated by the Brunauer–Emmett–Teller (BET) method. The pore size distribution was calculated by the Barrett–Joyner–Halenda (BJH) method.

### Electrochemical measurements

The working electrodes are fabricated by using LFP (or LFP/C) as the active materials, conductive carbon blacks (Super-P) and polyvinylidene fluoride (PVDF) binder in a weight ratio of 70:20:10. The slurry is coated on aluminum foil and dried in vacuum at 120 °C for 12 h. Electrochemical measurements are carried out via CR2025 coin type cell using lithium pellets as the counter electrode and the reference electrode, a 1 M solution of LiPF_6_ in ethylene carbon (EC)/dimethyl carbonate (DMC) (1:1 w/w) as electrolyte. The cells are assembled in an argon-filled glove-box. Cyclic Voltammetry (CV) measurements are carried out using a CHI 660D electrochemical workstation at a scanning rate of 0.1–2 mV s^−1^. Galvanostatic charge/discharge cycling is studied in a potential range of 2 V–4 V vs Li/Li^+^ with a multichannel battery testing system (LAND CT2001A). Electrochemical impedance spectra (EIS) are measured with an electrochemical workstation (Autolab PGSTAT 302N) in the frequency range from 100 KHz to 10 mHz.

## Additional Information

**How to cite this article**: Zhang, Q. *et al*. Engineering 3D bicontinuous hierarchically macro-mesoporous LiFePO_4_/C nanocomposite for lithium storage with high rate capability and long cycle stability. *Sci. Rep*. **6**, 25942; doi: 10.1038/srep25942 (2016).

## Supplementary Material

Supplementary Information

## Figures and Tables

**Table 1:** Kinetic parameters of the LFP and LFP/C electrodes.

Samples	R_e_ (Ω)	R_ct_ (Ω)	R_f_ (Ω)	CPE_1_ (F)	CPE_2_ (F)
LFP-10	3.8	1476.2	0.4	4.4 × 10^−5^	3.3 × 10^−14^
LFP/C-10	3.5	108.6	95.6	7.9 × 10^−6^	1.5 × 10^−3^
LFP/C-20	3.3	131.3	174.8	9.4 × 10^−6^	1.1 × 10^−3^
