# Supplementary material for: Engineering 3D bicontinuous hierarchically macro-mesoporous LiFePO4/C nanocomposite for lithium storage with high rate capability and long cycle stability
Source: Sci Rep. 2016 May 16;6:25942. doi: 10.1038/srep25942 (PMC4867577; doi:10.1038/srep25942)

Supplementary Information

Engineering 3D bicontinuous hierarchically macro-mesoporous $LiFePO_4$/C nanocomposite for lithium-ion battery with high rate capability and long cycle stability

Qian Zhang[1], Shao-Zhuan Huang[1], Jun Jin[1], Jing Liu[1], Yu Li[*1], Hong-En Wang[1], Li-Hua Chen[1], Bin-Jie Wang[4] and Bao-Lian Su[*1,2,3]

[1]Laboratory of Living Materials at the State Key Laboratory of Advanced Technology for Materials Synthesis and Processing, Wuhan University of Technology, 122 Luoshi Road, 430070, Wuhan, Hubei, China

[2]Laboratory of Inorganic Materials Chemistry (CMI), University of Namur, 61 rue de Bruxelles, B-5000 Namur, Belgium

[3]Department of Chemistry and Clare Hall, University of Cambridge, Cambridge, CB2 1EW, United Kingdom

[4]FEI company, Shanghai Nanoport, 399 Shenxia Road, 201210 Shanghai, China.

Correspondence and requests for materials should be addressed to Y. L. (yu.li@whut.edu.cn). Tel: (+86) 27 87855322. Fax: (+86) 27 87879468 or B.L.S. (bao-lian.su@unamur.be). Tel: (+32) 81 724531. Fax: (+32) 81 725414.

Figure S1. X-ray diffraction (XRD) patterns of the as-synthesized LFP-P.

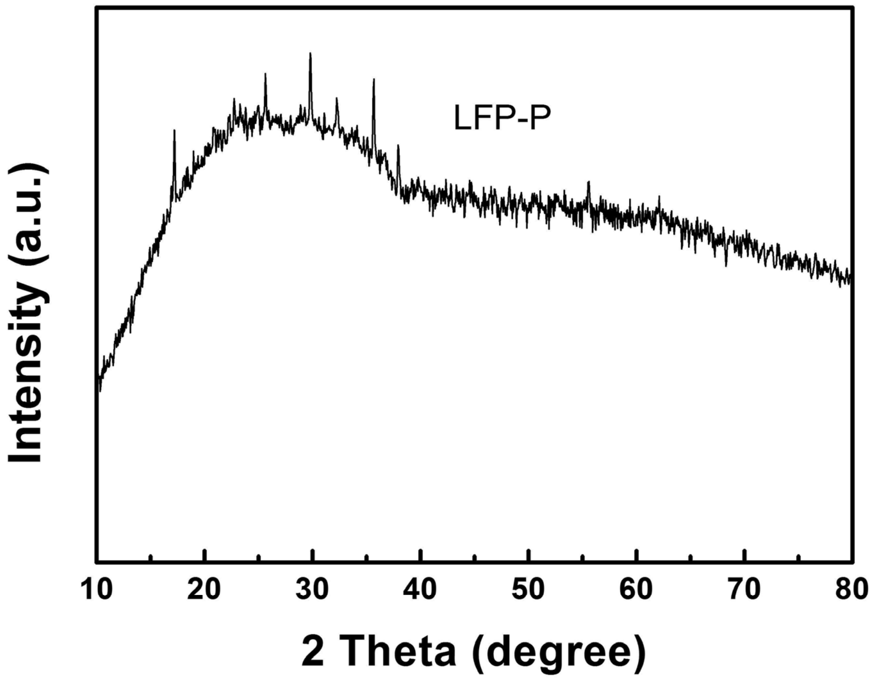

Figure S2. SEM images of the as-synthesized LFP-P.

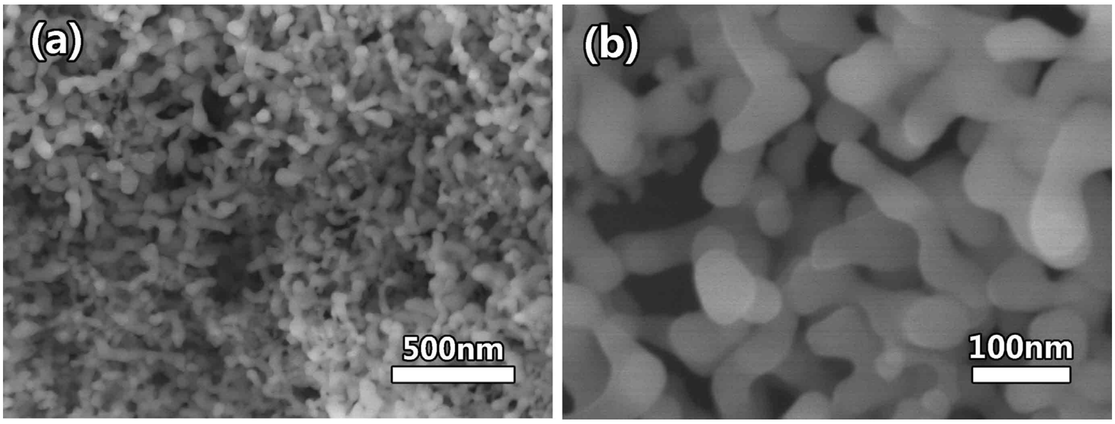

Figure S3. SEM image of the as-synthesized LFP/C.

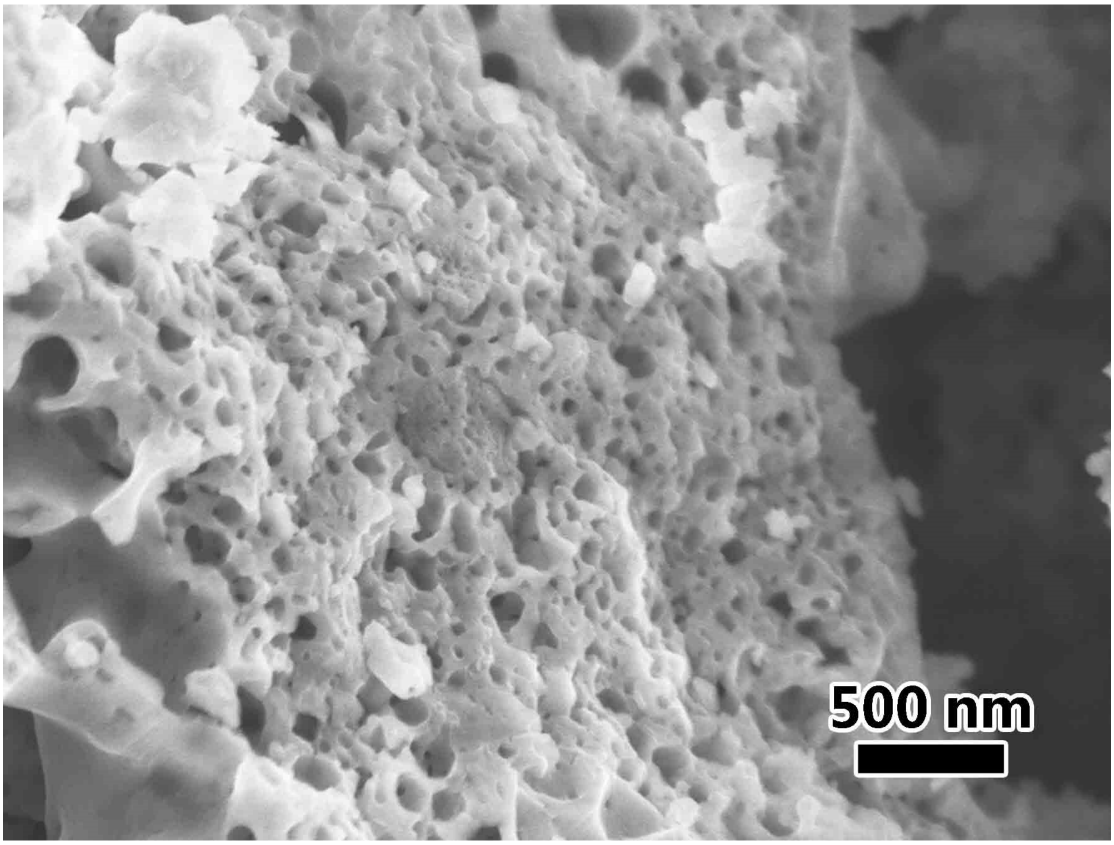

Figure S4. SEM images of the as-synthesized LFP/C.

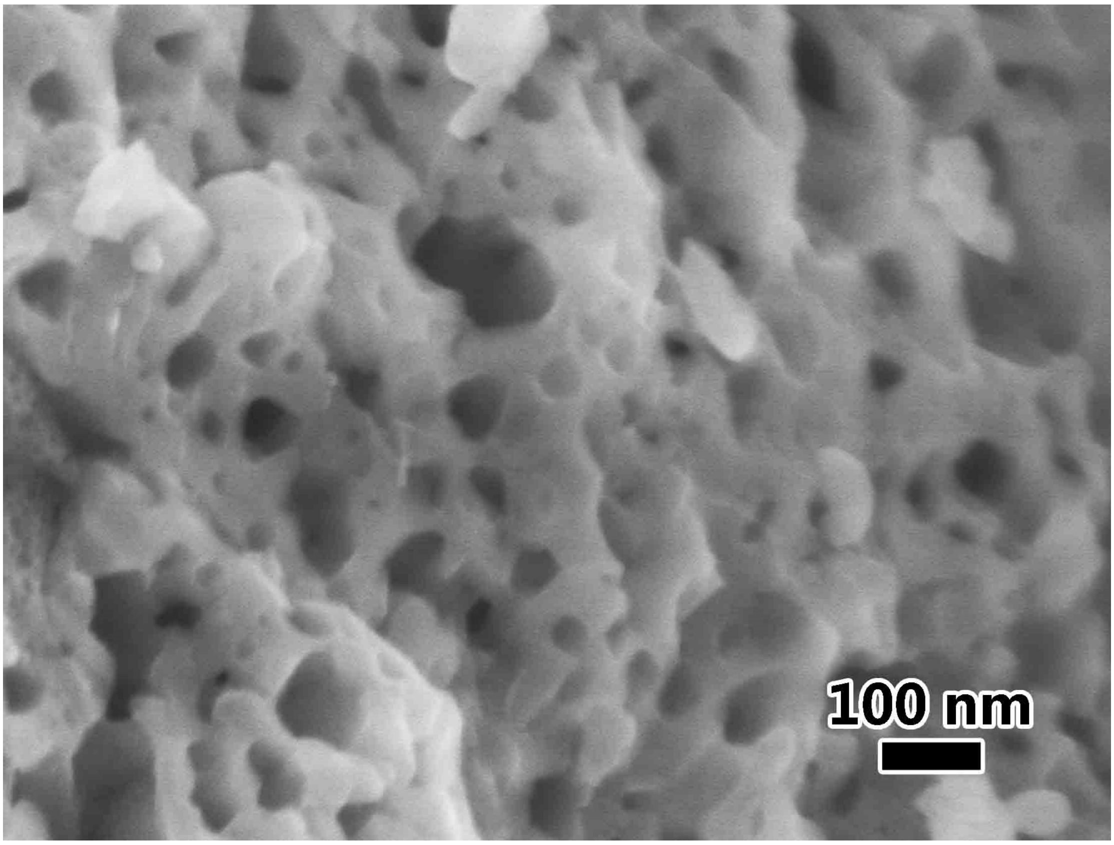

Figure S5. SEM image of the as-synthesized LFP/C.

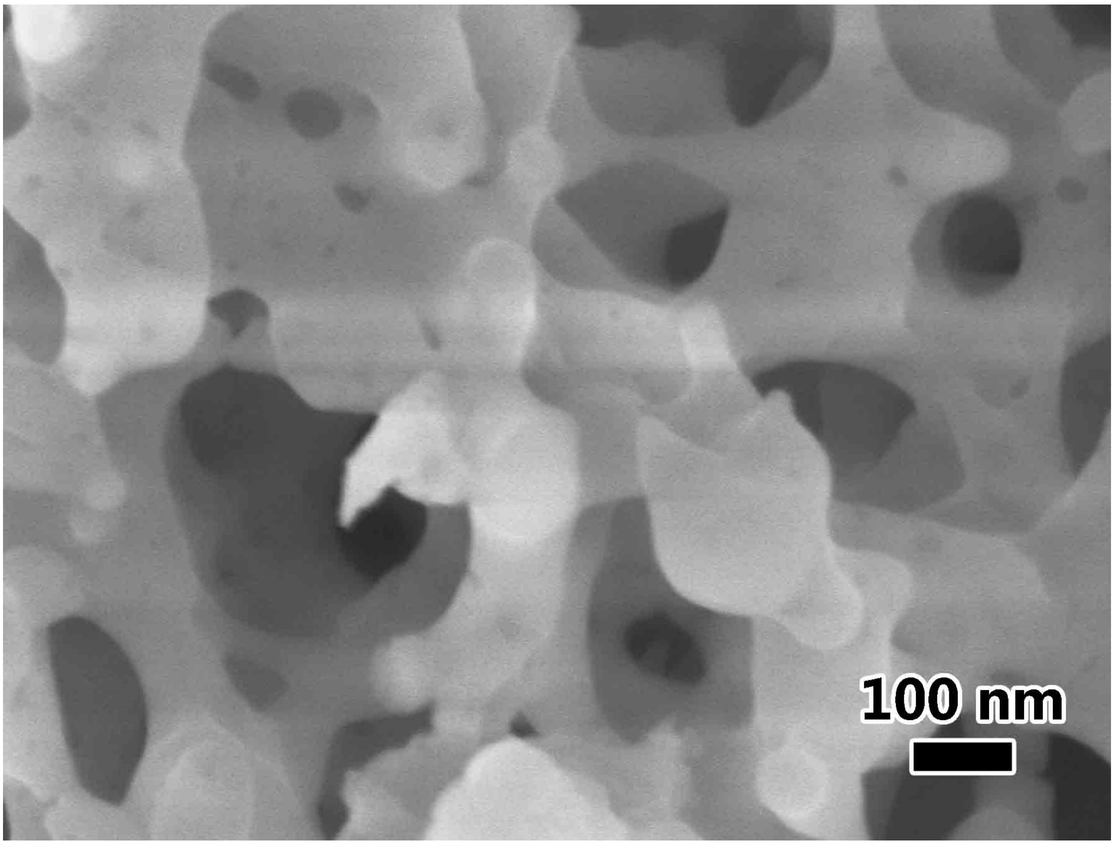

Figure S6. (a) TEM and (b) HRTEM images of LFP.

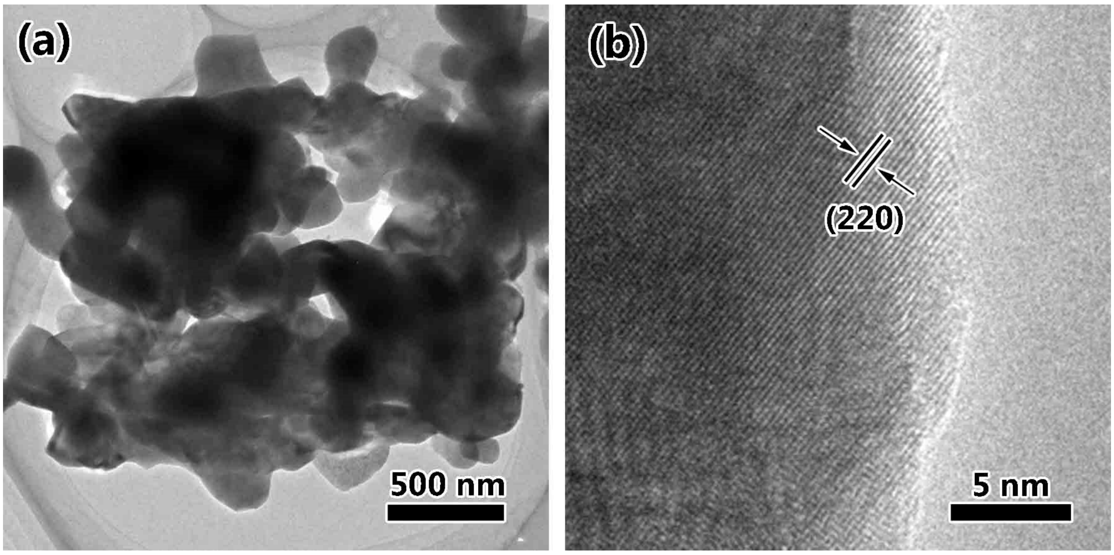

Figure S7. TEM images of the as-synthesized LFP/C.

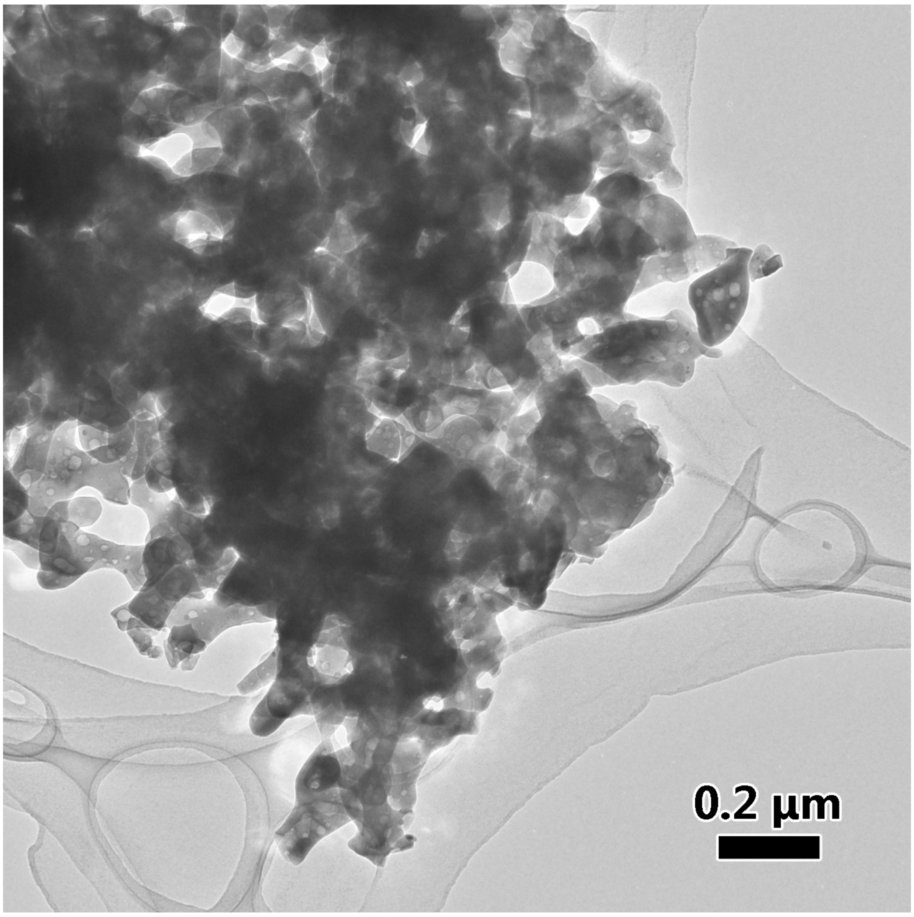

Supplement: Supplementary Information [file srep25942-s1.pdf]
